# How Warmth Appeal Affects Persuasion: The Moderating Role of Brand Concepts

**DOI:** 10.3389/fpsyg.2022.831373

**Published:** 2022-03-30

**Authors:** Fei Jin, Jixuan Zhang, Banggang Wu, Xiaodong Zhu

**Affiliations:** ^1^Department of Marketing and E-Commerce, Sichuan University, Chengdu, China; ^2^International Tourism College, Hainan University, Haikou, China; ^3^Department of Sports, Tsinghua University, Beijing, China

**Keywords:** advertising, warmth appeal, warmth perception, competence perception, brand concepts

## Abstract

In practice, more and more companies are using warmth appeals in their advertisements, but not all warmth appeals can bring the expected results. Grounded in social perception, we propose that consumers’ inferences and behavioral intentions stemming from warmth appeals in advertising are moderated by brand concepts. Specifically, warmth appeal decreases competence inferences and, in turn, behavioral intentions toward the self-enhancement brands. However, it increases warmth inferences and, in turn, behavioral intentions toward self-transcendence brands. We tested our hypotheses through two experimental studies. Experiment 1 demonstrated that for self-enhancement brands, warmth appeals in advertisements decreased brand attitudes and purchase intentions; for self-transcendence brands, warmth appeals in advertisements increased brand attitudes and purchase intentions. Experiment 2 showed further evidence to the proposed effect and tested the mediating effects of warmth perception and competence perception. This research provides significant implications for advertising strategies.

## Introduction

Advertising is an important way for companies to publicize product information, convey brand value, build brand image, and highlight product or brand features (Liu-Thompkins, 2019). It influences or even directly determines consumers’ purchase decision. For that reason, companies spend a lot of money on advertising every year, constantly exploring appropriate advertising strategies in an attempt to influence consumers’ perceptions and attitudes (Royne et al., 2012; Aaker and Biel, 2013). A common strategy used by companies and brands is to show warmth and tenderness in their advertisements (Choi et al., 2016; Kolbl et al., 2020; Zhang et al., 2020). For example, the 25th anniversary ad campaign of Safeguard used a warm tone to depict details of a mother’s and daughter’s daily life, with melodious background music to tell the story of two generations growing up with the brand. SAIC-Volkswagen launched a warm television advertisement “Companionship” for its SUVs, recording the wonderful experience of family travel from the perspective of a pet dog. Both these advertisements have received widespread praise. As advertisers increasingly seek to strengthen relationships with consumers, commonsense would hold that promoting warmth can enhance brand evaluations and build a relationship with consumers by fostering positive feelings that imbue the brand with a caring and generous image.

However, in practice, the warmth appeal in advertisements does not always bring positive effects (Li et al., 2019). For example, China Merchants Bank’s tender ad “No matter how big the world is, it’s no bigger than a plate of scrambled eggs with tomatoes” has been widely discredited. Many consumers find the motherly love story related to cooking emotional, but it is difficult to associate with China Merchants Bank. Moreover, the ad content is too sentimental, and the bank is suspected of avoiding core value transmission by playing an emotion card.

Academics have carried out a rich discussion on the influence of warmth appeal in advertising, arguing that warm ads containing emotional elements can, for instance, enhance the emotional connection between brands and consumers, draw people closer to brands (Fournier and Alvarez, 2012), influence consumers’ cognitive evaluation process of ads (Li and Miniard, 2006), and enhance the perception of brand sincerity (Aaker and Bruzzone, 1981). Existing studies, however, have been limited to the positive effects of warmth appeal and have not explored in more detail when they can be negative. This possibility is important for companies and advertising decision-makers.

Psychological studies have shown that people’s evaluation of warmth appeal can be influenced by other external cues (Srull and Wyer, 1979; Carlston and Mae, 2006; Uleman et al., 2008). In the marketing context, warmth appeal is closely related to brand image (Kunkel et al., 2019). As every brand has a relatively stable brand concept, how is warmth appeal in advertising influenced by the brand’s inherent concept? Existing studies have not provided clear answers. Furthermore, existing research has not systematically investigated the mechanisms underlying the impact of warmth appeal in advertising. Based on social perception, this paper examines how warmth appeal affects consumers’ perceptions of warmth and competence, which in turn influence their brand attitude and purchase intention. Such investigation is a valuable supplement to existing research.

## Theoretical Framework

### Warmth Appeal in Advertising

Warmth appeal in advertising refers to “the positive, gentle, fleeting emotional, and psychological arousal that people feel from an advertisement, usually from the love, family, or friendship” (Aaker et al., 1986). It reflects traits such as being caring or helpful (Bolton and Mattila, 2015). In our study, warmth appeal in advertising refers to content promotion related to the good intention of the brand, using gentle strategies such as love and friendliness, care and communication. For example, aiming to embody the “trust” and “protection” that it conveys, Alipay launched a commercial in 2017 that cuts to four scenes of life, accompanied by a song by Li Zhi, telling a story of Alipay and life. Alipay’s official microblogging site released a video with the text “There are a hundred flavors of life, we’re there for you, enjoy what you like, share the pain, no matter what, live your life to the fullest.”

Previous research suggests that the inclusion of warmth appeal in advertising can have a positive effect, which is the halo effect (Asch, 1946; Nisbett and Wilson, 1977). Zawisza and Pittard (2015) argue that warmth appeal can increase favorable attitudes and purchase intention for low-involvement products, but non-warmth appeal can have a positive effect for high-involvement products. On online platforms, warmth-oriented service recovery messages enhance observers’ service perceptions more than competence-oriented messages (Huang and Ha, 2020). Warmth appeal (vs. non-warmth appeal) can improve perceptions of brand sincerity (Aaker et al., 2004).

According to existing research, warmth and non-warmth appeal in advertising are two different types of approaches that influence consumer preferences and willingness to purchase. Warmth appeal in advertising, as an external and immediate stimulus, can stimulate cognitions about warmth-related issues. However, consumers have an inherent and solid, meaningful system to form perceptions and expectations about external things based on their past experiences (Cuddy et al., 2004). In the case of brands, consumers also have a relatively stable cognition. This study investigates the impact of warmth appeal with different brand concepts.

### Brand Concepts

Brand concept refers to the “unique, abstract meanings” associated with a brand. These unique, abstract meanings are derived from brand attributes, benefits, and other marketing efforts that can be translated into higher-level concepts (Park et al., 1991). It includes both concrete (what the brand actually does) and abstract (the way people think about the brand in the abstract) aspects of the brand (Monga and John, 2010). Torelli et al. (2012a) suggest that brand philosophy can be divided into two corresponding dimensions: self-enhancement and self-transcendence. The former refers to the pursuit of social status and prestige as well as control over others or resources, and emphasizes the demonstration of personal success through competitiveness based on social standards. The latter emphasizes understanding, gratitude, tolerance, and protection of human interests, and also includes the value of protecting the environment (Torelli et al., 2012b).

Extant research suggests that consumers are influenced by brand concepts when they process a company’s marketing messages. Torelli et al. (2012a) found that luxury brands with a self-enhancement brand concept (vs. control group) had lower consumer evaluations when they promote their corporate social responsibility (CSR) messages, because the self-enhancement concept and concern for the well-being of others conveyed in the message are in conflict. Similarly, in a global branding context, an empirical study based on respondents from eight countries showed that a brand is more acceptable if the brand concept is compatible or coordinated with local cultural value (Torelli et al., 2012b).

As brand concept is an existing and relatively stable perception of the brand, consumers are faced with two possibilities for warmth appeal: consistent with the brand concept or inconsistent with it. Consumers tend to react more positively when relevant activities are consistent with their expectations (Aggarwal, 2004), but they tend to do deeper thinking when relevant information is inconsistent with their expectations (Lynch and Schuler, 1994). How then do consumers interpret the relationship between warmth appeal and brand concept? From a cognitive perspective, consumers’ interpretation of warm ads is a process that how they perceive the information. In our study we use social perception to explore underlying mechanisms of the influence of warmth appeals.

### Warmth Appeal and Social Perception

Warmth and competence are two basic dimensions of social perception (Torelli et al., 2012b). The former describes how people behave socially, while the latter refers to how they behave in terms of effectiveness (Wang et al., 2017). Warmth primarily includes perceptions and judgments of generosity, kindness, honesty, sincerity, helpfulness, trustworthiness, and consideration for others, while competence refers mainly to perceptions of confidence, effectiveness, diligence, ability, professionalism, and competitiveness (Judd et al., 2005; Yzerbyt, 2008). At a higher level, showing warmth implies an orientation toward others as well as motivation to follow moral norms; demonstrating competence implies of the capacity to achieve one’s intention (Cuddy et al., 2008).

Although warmth and competence are originally used to assess people’s perceptions and evaluations of the image of others, they are often used to “personify” an organization’s image. Aaker et al. (2010) show that non-profit organizations bring a stronger perception of warmth, and for-profit organizations bring a stronger perception of competence. On the one hand, the warmth appeal in advertising can bring about a priming effect which triggers the perception of warmth. On the other hand, warmth appeal may provide an important cue for people’s judging the competence dimension of a product. Thus, how warmth appeal affects people’s warmth perception and competence perception and the relationship between them invite further investigation.

### Hypothesis Development

**FIGURE 1:**
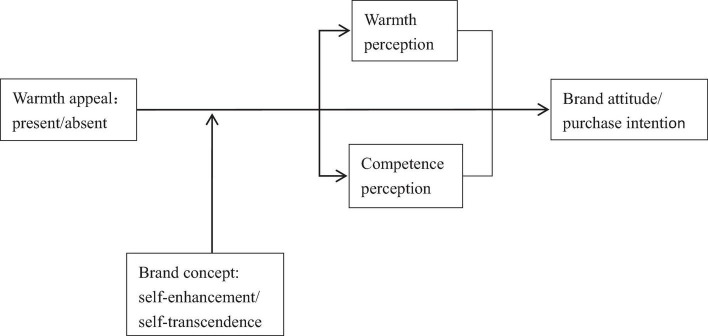
Research framework.

Consumers do not accept external information blindly but reason for it. Reasoning refers to the means by which people process, infer, and draw conclusions from the visible and available information (Srull and Wyer, 1979). Reasoning may be a purposeful, conscious aspect of explicit cognitive processing or an automatic, unconscious part of implicit cognitive processing (MacKenzie et al., 1986; Brambilla et al., 2010). Reasoning is very common in consumer behavior research as well as in everyday life, such as people’s reasoning about the relationship between products and prices, and the relationship between appearance and ability. People’s reasoning is influenced both by external situational factors and irrelevant factors. In the reasoning process, if target characteristics fit with desired traits, it will produce positive results. If there is no such fit, the results may be negative. We argue that consumers’ reasoning about the warmth of an ad is influenced by its match with the characteristics of the product or brand (Figure 1).

Self-enhancement refers to the pursuit of social status and prestige, emphasizing the development of individual capabilities and success. Brands with the concept of self-enhancement are generally exclusive in nature, which is a way to demonstrate the owner’s distinctive identity and social status (Torelli et al., 2012b). Such brands are generally of high quality and expensive, such as LV, Gucci, BMW, and consumers expect to make an association with them as superior brands. Self-transcendence means looking beyond the interests of the individual and caring about the welfare of the wider public, with an emphasis on understanding, caring, and inclusivity. Such brands generally portray an approachable brand image. Toms Shoes, for example, when Toms Shoes sells a pair of shoes, it donates a pair to a child in Africa (ONE FOR ONE). Thus, consumers’ expectations for self-enhancement brands focus more on traits related to competence and competition such as good quality and distinctive identity, while their expectations for self-transcendence brands focus more on emotional traits such as approachability and public well-being. In their reasoning, people will evaluate and judge whether the brand or product matches their expectations based on their inherent perceptions.

For self-enhancement brands, warmth advertising appeal (vs. no warmth advertising appeal) is inconsistent with consumers’ expectations, resulting in an incongruent perception and deeper cognitive processing (Lynch and Schuler, 1994). In this situation, an ad’s warmth appeal message has a strong reminding effect, and consumers are more likely to associate the lack of a warmth cue with the self-enhancement brand, reducing people’s ability to perceive it. Previous research has shown that people have a negative bias toward warmth cues, and the negative effects of the absence of warmth are much greater than the positive effects of the presence of warmth (Cuddy et al., 2007). In short, warmth appeal is largely incompatible with a brand concept that emphasizes self-enhancement, and it can reduce the competence perception of the self-enhancement brand concept.

In contrast, for a self-transcendence brand, an ad’s warmth appeal is in line with consumers’ expectations and thus brings positive responses (Judd et al., 2005). First, the self-transcendence brand focuses on public welfare and emphasizes caring and inclusivity. People’s expectations of this brand lead them pay more attention to warmth cues (vs. competence cues). At the same time, this focus on warmth cues decreases their attention to competence; therefore, competence reasoning is less affected when the warmth appeal is present in an ad. Second, because consumers accept and recognize the warmth appeal in the ad, purchasing a self-transcendence brand indicates that the consumer is approachable. Therefore, we propose two hypotheses.

***H1a:** The effect of warmth appeal on inferred competence is more unfavorable under self-enhancement brand concept than under self-transcendence brand concept.*

For self-enhancement brands, competence (vs. warmth) information has greater diagnosticity for consumers, and people focus more on the competence dimension and relatively ignore the warmth dimension. Further, two opposing inferences lead people to a neutral net effect on warmth perceptions for self-enhancement brands. On the one hand, people believe that the competence dimension of the self-enhancement brand is dominant which leads to a positive effect. On the other hand, this positive effect is influenced by the negative inference that brands which are supposed to be superior are exhibiting warmth, which reduces the positive perception. For self-transcendence brands, the warmth cue receives more attention, and the warmth appeal in the ad coincides with this cue, and the consistency enhances the perception of warmth. Thus:

***H1b:** The effect of warmth appeal on inferred warmth is more favorable under self-transcendence brand concept than under self-enhancement brand concept.*

Further, because people’s warmth perception and competence perception affect their intention and behavior (Lynch and Schuler, 1994), we argue that warmth perception and competence perception have different effects on consumers’ brand attitudes and purchase intention according to brand concept condition. Based on the previous discussion, we propose that:

***H2:** Consumer attitudes toward companies using warmth appeal in ads (vs. no warmth appeal) decrease for self-enhancement brand concept but increase for self-transcendence brand concept.*

## Research Overview

We examined our hypotheses using two experiments. Experiment 1 preliminarily demonstrated the impact of warmth appeal in advertising on consumers (brand attitude/purchase intention) in different brand concept contexts. Experiment 2 replicated the results of Experiment 1 with a different product, and further demonstrated the mediating role of consumer perceptions of competence and warmth. Across studies, we systematically applied the same sample filtering criteria.

## Experiment 1

Experiment 1 first demonstrated how warmth appeal in advertising influences consumer judgments and decisions in different brand concept contexts. When companies use warmth appeal (vs. absence of warmth appeal) in advertising, for self-transcendence brands the effect on consumers’ attitude and purchase intention is positive, while for self-enhancement brands the effect on consumers’ attitude and purchase intention is negative. To reduce potential confounding caused by inherent branding, we used a fictitious car brand to validate our hypothesis.

### Pre-test

To select the appropriate experimental material for this study and exclude the confusing effects of product classification, product involvement, or differences in demand, a pre-test was conducted prior to the formal experiment. From interviewing five doctoral students and two professors in marketing, we selected cars as the experimental material and manipulated an advertising slogan for a fictitious car brand to present different brand concepts.

We then emailed an experiment link to 40 MBA students (15 females, M_age_ = 30.55, SD = 4.85), each of whom received a $3 payment upon completion of the experiment. The participants were randomly assigned to two groups and asked about their brand perception. Due to the halo effect of the more attractive product, we wanted to make sure that there was no difference in the appeal of the product between the two contexts. We also measured their perception of safety (“To what extent do you think the car is safe?” 1 = very unsafe, 7 = very safe). Finally, we measured their purchase intention.

**FIGURE 2:**
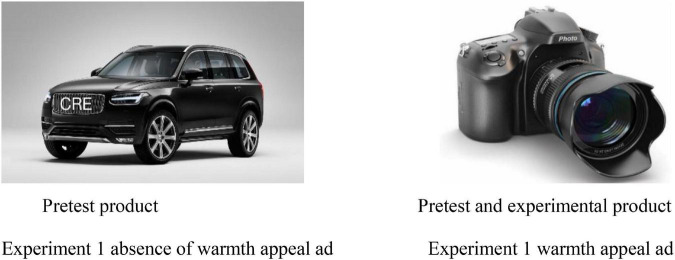
Pictures of the experimental materials in Experiments 1 and 2. Reproduced with permission from carsales and Saint-Louis Studio (image sources, car: https://www.carsales.com.au/volvo/xc90/price/2019/; camera: https://clubofmozambique.com/business-directory/saint-louis-studio/).

We randomly divided the participants into two groups and presented them with pictures of cars (see Figure 2), telling them to imagine that CRE, a world-renowned car manufacturer, had launched a luxurious/eco-friendly car in the first half of the year. The self-enhancement brand concept was invoked with “Elite technology, bring luxury experience. The top configuration is a symbol of your wealth and status. CRE, enjoy extraordinary driving”; The self-transcendence brand concept was indicated by “Advanced technology, bring friendly experience, attentive service, is the guarantee of your attentiveness and comfort. CRE, enjoy the pleasure of driving.” After that, they were told that every brand has a brand concept which represents the value and attitude of the enterprise. A self-enhancement brand emphasizes the pursuit of social status and prestige and promotes personal success through competitiveness according to social standards; a self-transcendence brand emphasizes understanding, appreciation, tolerance, and protection of human interests. They then rated the brand concept based on the products and descriptions they saw (1 = self-transcendence, 7 = self-enhancement). Higher scores indicated that participants perceived the brand more as self-enhancement and, conversely, less as self-transcendence (Monga and John, 2010).

The results showed that our manipulation was successful [M_self–transcendence_ = 3.15, M_self–enhancement_ = 5.30, *F*_(1,38)_ = 20.73, *p* < 0.01]. Participants perceived the brand concepts as intended. There was no difference in attractiveness between cars (M_self–transcendence_ = 5.15, M_self–enhancement_ = 5.60, *ns*). Nor was there a difference in the perception of the safety of the car (M_self–transcendence_ = 4.25, M_self–enhancement_ = 4.40, *ns*), or in the subjects’ purchase intention (M_self–transcendence_ = 5.35, M_self–enhancement_ = 5.75, *ns*).

### Materials and Methods

In Experiment 1, we used a 2 (warmth appeal: presence vs. absence) ×2 (brand concept: self-enhancement vs. self-transcendence) between-subjects design to test our hypotheses. We first informed participants that the CRE brand had recently considered launching an advertisement. The warmth appeal was reflected in two aspects: (1) the overall tone, including soft colors and a slogan that read, “We are together for a pleasant journey” (Aaker et al., 1986); and (2) a picture of three families interacting with each other (Brambilla et al., 2010). In comparison, the ad without warmth appeal was in a cool grayish tone with the slogan “Driving on the road, CRE goes with you.”

### Procedure

Similar to the pre-test, we emailed the link for the experiment to 180 MBA students at one university, but 8 of them failed our attention test, leaving a valid sample of 172 (80 women, M_age_ = 34.6, SD = 1.30). Before the start of the experiment, we told participants that the study concerned marketing a car brand, and there were no right or wrong answers. They were then randomly assigned to one of the four condition.

We then informed participants of the CRE brand and told them that it had recently considered launching an advertisement. Next, we measured their perceptions of the ad using four descriptors: to what extent do you perceive the ad you saw as cozy, warm, conveying a sense of warmth, and full of affection (α = 0.84) (Zawisza and Pittard, 2015). We also assessed their attitudes toward the brand: bad-good, unfavorable-favorable (α = 0.91) (Aaker et al., 2012) and willingness to buy (totally unlikely-very likely). In addition, we measured the participants’ mood after viewing the ad (very negative-very positive). All ratings were on 7-point scales. To conceal the purpose of the experiment further, we asked them to list their favorite car brands. Finally, we collected demographic information.

### Results

#### Manipulation Test

First, we tested whether the manipulation of warmth appeal was successful. The results showed a significant difference between the two ads [*F*_(1,170)_ = 40.50, *p* < 0.01]. Compared with the group without warmth appeal (M = 3.97, SD = 1.38), participants in the warmth appeal group (M = 5.15, SD = 1.05), perceived more warmth. There was no difference in the mood of the two groups after watching the ad [M_warmth appeal_ = 4.79, M_absence of warmth appeal_ = 4.63, *F*_(1,170)_ = 0.71, *p* = 0.40, *ns*].

#### Brand Attitude

We first conducted a one-way ANOVA on brand attitude (Experiments 1 and 2, see Table 1; α = 0.89). The results showed that the main effect on brand concept was significant [*F*_(1,168)_ = 5.39, *p* < 0.05, η*^2^* = 0.03], and the main effect on warmth appeal was not significant [*F*_(1,168)_ = 0.09, *p* = 0.76, *ns*]. The interaction effect between warmth appeal and brand concept was significant [*F*_(1,168)_ = 6.99, *p* < 0.01, η*^2^* = 0.04]. Planned contrast analysis showed that when in the self-enhancement brand concept condition, people rated the brand significantly more favorably when there was no warmth appeal than when there was warmth appeal [M_warmth appeal_ = 5.26, SD = 1.04, M_absence of warmth appeal_ = 5.68, SD = 1.01, *t*_(168)_ = 2.08, *p* < 0.05]. Under the self-transcendence brand concept condition, warmth appeal led people to rate the brand higher than those without warmth appeal. This effect was marginally significant [M_warmth appeal_ = 5.97, SD = 0.72, M_absence of warmth appeal_ = 5.63, SD = 0.97, *t*_(168)_ = 1.66, *p* = 0.07]. Further, when warmth appeal was present, brand attitude under the self-transcendence brand concept was significantly higher than under the self-enhancement brand concept condition [M_self–transcendence_ = 5.97, SD = 0.72, M_self–enhancement_ = 5.26, SD = 1.04, *t*_(168)_ = 3.42, *p* < 0.01]; when warmth appeal was absent, there was no significant difference in brand attitude between the self-transcendence and self-enhancement brand concepts [M_self–transcendence_ = 5.63, SD = 0.97, M_self–enhancement_ = 5.68, SD = 1.01, *t*_(168)_ = 0.22, *p* = 0.83, *ns*].

**TABLE 1:** Relevant constructs and measures in Experiment 2.

Constructs	Terms	Cronbach α	References
Attitude toward ad	To what extent do you perceive the ad you saw as cozy/warm/conveying warmth/full of affection?	0.90	Zawisza and Pittard, 2015
Warmth perception	I think OLM is friendly/humane/concerned about consumer rights.	0.89	Scott et al., 2013
Competence perception	I think OLM is competitive/efficient/in the lead position.	0.91	
Brand attitude	Bad–good, Unfavorable–favorable	0.87	MacKenzie et al., 1986
Purchase intention	Willingness to buy		

#### Purchase Intention

**FIGURE 3:**
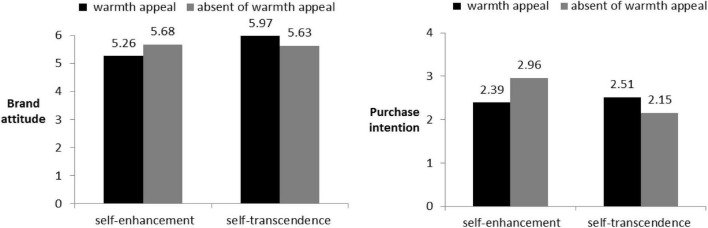
Brand attitude and purchase intention in Experiment 1.

Next, we conducted a one-way ANOVA on purchase intention. The results showed that neither the main effect of warmth appeal [*F*_(1,168)_ = 3.06, *p* = 0.08, *ns*] nor the main effect of brand concept [*F*_(1,168)_ = 0.27, *p* = 0.61, *ns*] was significant, but the interaction effect between warmth appeal and brand concept was significant [*F*_(1,168)_ = 5.58, *p* < 0.05]. We then conducted a planned contrast analysis. Under the self-enhancement brand concept, the presence or absence of warmth appeal in the ad did not affect people’s purchase intention [M_warmth appeal_ = 2.39, SD = 1.53, M_absence of warmth appeal_ = 2.51, SD = 1.14, *t*_(168)_ = 0.43, *p* = 0.67, *ns*]. In the self-transcendence brand concept condition, people had higher purchase intention after seeing the warmth appeal [M_warmth appeal_ = 2.96, SD = 1.27, M_absence of warmth appeal_ = 2.15, SD = 1.16, *t*_(168)_ = 2.91, *p* < 0.05]. Further, when the warmth appeal was present, there was no significant difference between purchase intention under the self-transcendence and self-enhancement brand concepts [M_self–transcendence_ = 2.51, SD = 1.14, M_self–enhancement_ = 2.39, SD = 1.53, *t*_(168)_ = 0.61, *p* = 0.55, *ns*]. When warmth appeal was present, purchase intention was significantly lower under the self-transcendence brand concept than under the self-enhancement brand concept [M_self–transcendence_ = 2.15, SD = 1.16, M_self–enhancement_ = 2.96, SD = 1.27, *t*_(168)_ = 3.64, *p* < 0.01]. The results are shown in Figure 3.

Experiment 1 provides preliminary evidence for our hypothesis: for different brand concepts (self-enhancement vs. self-transcendence), the use of warmth appeal (vs. absence of warmth appeal) in a firm’s advertising has different effects. For self-transcendence brands, warmth appeal (vs. absence of warmth appeal) had positive effects on people’s attitude and purchase intention; for self-enhancement brands, warmth appeal (vs. absence of warmth appeal) had negative effects on people’s attitude and purchase intention. In Experiment 1, we used an automobile as the experimental material, and people may pay more attention to the strength-related attribute of that product than others. If we used other product categories for which a strength-related attribute is not obvious, would the effect still occur? Further, if brand concept still moderated the impact of warmth appeal, what would be the underlying mechanism?

## Experiment 2

Experiment 2 had two main purposes: first, to assess our hypotheses in a different product and brand context; and second, to examine the mediating effect of competence perception/warmth perception.

### Pre-test

As in Experiment 1, we first a determined a product. We chose a camera as our experimental material. We presented a picture of a camera (see Figure 2) with different descriptions. For the self-enhancement brand concept, the description read “OLM company always adheres to the brand concept of technology into the future. They are constantly improving their technology, and have been enhancing the consumer’s taste for decades. Flawless, everything is under your control.” The self-transcendence brand concept description was “OLM always insists on the brand concept of technology improving life. They have been improving technology and enhancing consumer welfare for decades. Perfection, for every moment of your life.” After being informed of the definition of brand concept, the participants were asked to rate the brand concept on a 7-point scale (1 = self-transcendence, 7 = self-enhancement) based on the products and descriptions they saw (Goldsmith et al., 2000).

Forty participants from MTurk (19 females, M_age_ = 36, SD = 2.65) participated in our pre-test. They were randomly assigned to two groups, and paid $0.2 after completing the experiment. The results indicated that our manipulation was successful [M_self–transcendence_ = 3.55, M_self–enhancement_ = 5.73, *F*_(1,38)_ = 22.99, *p* < 0.01] and there was no significant difference in attractiveness between the two brands [M_self–transcendence_ = 5.85, M_self–enhancement_ = 5.90, *F*_(1,38)_ = 0.02, *p* = 0.89, *ns*], or in participants’ purchase intention [M_self–transcendence_ = 5.60, M_self–enhancement_ = 5.55, *F*_(1,38)_ = 0.02, *p* = 0.90, *ns*].

### Materials and Methods

In Experiment 2, we again used a 2 (warmth appeal: presence vs. absence) × 2 (brand concept: self-enhancement vs. self-transcendence) between-group design to test our hypothesis. We first told the participants that the camera equipment company OLM was going to launch an advertising campaign and wanted consumers’ evaluation. The ad for the warmth appeal group was “Clear image quality, reliable image stabilizer, excellent visibility. Warm company freezes the beauty”; the absence of warmth appeal group message was “Clear image quality, reliable image stabilizer, excellent visibility. Touch the shutter to record the moment.”

### Procedure

Two hundred participants from MTurk participated in the experiment for $0.6 compensation. Five failed attention test questions, leaving a valid sample of 195 (117 females, M_age_ = 41.61, SD = 2.38). Before the experiment began, we informed participants that this was a study of camera preference and there were no right or wrong answers. After that, they were randomly assigned to one of the four conditions. First, participants read the information about OLM brand and its ads. Second, they rated their attitude toward the ad, warmth perception and competence perception, and brand-related outcome variables (brand attitude and purchase intention), as detailed in Table 1. All measures used 7-point scales. We also measured their knowledge about the camera. Finally, demographic information was collected.

### Results

#### Manipulation

First, we tested whether the manipulation of warmth appeal was successful. The results showed a significant difference between the two ads [*F*_(1,193)_ = 41.33, *p* < 0.01]. Compared to the without warmth appeal group (M = 4.04, SD = 1.55), participants in the warmth appeal group (M = 5.45, SD = 1.51) perceived more warmth.

#### Competence Perception

A one-way ANOVA was conducted for competence perception. The results showed that the main effect of neither warmth appeal [*F*_(1,191)_ = 3.31, *p* = 0.07, *ns*] nor brand concept [*F*_(1,191)_ = 2.76, *p* = 0.10, *ns*] was significant, but the interaction effect of warmth appeal with brand concept was significant [*F*_(1,176)_ = 14.61, *p* < 0.01]. Specifically, when in the self-enhancement brand concept condition, the warmth appeal made people’s perception of competence lower [M_warmth appeal_ = 3.90, SD = 1.70, M_absence of warmth appeal_ = 5.18, SD = 1.27, *F*_(1,191)_ = 14.36, *p* < 0.01], whereas in the self-transcendence brand concept condition, perception of competence was unaffected (M_warmth appeal_ = 4.39, SD = 1.30, M_absence of warmth appeal_ = 3.93, SD = 1.69, *ns*).

#### Warmth Perception

A one-way ANOVA was conducted for warmth. The results showed that the main effect of warmth perception [*F*_(1,191)_ = 4.24, *p* < 0.05] and the interaction effect of warmth perception and brand concept [*F*_(1,191)_ = 6.57, *p* < 0.05] were significant. The main effect of brand concept was not significant [*F*_(1,191)_ = 2.26, *p* = 0.13, *ns*]. Specifically, for the self-enhancement concept, the warmth appeal did not affect people’s perception of warmth (M_warmth appeal_ = 3.92, SD = 1.54, M_absence of warmth appeal_ = 4.03, SD = 1.71, *ns*). For the self-transcendence concept, warmth appeal enhanced people’s warmth perception [M_warmth appeal_ = 4.80, SD = 1.44, M_absence of warmth appeal_ = 3.80, SD = 1.34, *F*_(1,191)_ = 11.98, *p* < 0.01].

#### Brand Attitude

The results of a one-way ANOVA showed that the main effects of brand concept [*F*_(1,191)_ = 2.90, *p* = 0.09, *ns*] and warmth appeal [*F*_(1,191)_ = 0.08, *p* = 0.78, *ns*] were not significant. However, the interaction effect of brand concept and warmth appeal was significant [*F*_(1,191)_ = 9.28, *p* < 0.01]. Further, planned contrast analysis showed that in the self-enhancement brand concept condition, compared to the condition with warmth appeal, people rated the brand significantly higher when there were no warmth appeal in the ad [M_warmth appeal_ = 4.69, SD = 1.48, M_absence of warmth appeal_ = 5.46, SD = 1.32, *F*_(1,191)_ = 4.99, *p* < 0.05]. In the self-transcendence brand concept condition, warmth appeal led people to rate the brand significantly higher than did without warmth appeal [M_warmth appeal_ = 5.00, SD = 1.35, M_absence of warmth appeal_ = 4.36, SD = 1.56, *F*_(1,191)_ = 4.30, *p* < 0.05].

#### Purchase Intention

**FIGURE 4:**
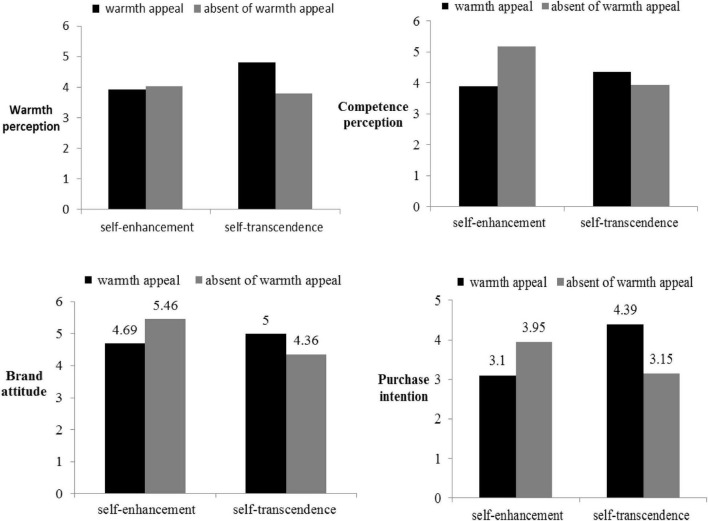
Warmth perception, competence perception, brand attitudes and purchase intention in Experiment 2.

Next, we conducted a one-way ANOVA on purchase intention. The results showed that neither the main effect of warmth appeal [*F*_(1,191)_ = 0.78, *p* = 0.38, *ns*] nor the main effect of brand concept [*F*_(1,191)_ = 1.22, *p* = 0.27, *ns*] was significant, but the interaction effect between warmth appeal and brand concept was significant [*F*_(1,191)_ = 22.21, *p* < 0.01]. Then, we conducted a planned contrast analysis. When participants were exposed to a self-enhancement brand image, both the presence and absence of warmth appeal in the ads influenced people’s purchase intention, although warmth appeal increased purchase intention to a greater extent [M_warmth appeal_ = 3.10, SD = 1.51, M_absence of warmth appeal_ = 3.95, SD = 1.65, *F*_(1,191)_ = 6.61, *p* < 0.05]. In the self-transcendence brand concept condition, people showed higher purchase intention when they were exposed to warmth appeal [M_warmth appeal_ = 4.39, SD = 1.51, M_absence of warmth appeal_ = 3.15, SD = 1.46, *F*_(1,191)_ = 17.58, *p* < 0.01; Figure 4].

#### Mediation

We followed the mediation effect analysis procedure proposed by Zhao et al. (2010), and conducted a Bootstrap test in reference to the multi-category categorical independent variable model proposed by Preacher and Hayes (2008), with warmth appeal as the independent variable; brand concept as the moderated variable; brand attitude as the dependent variable; and warmth perception and competence perception as mediated variables. We selected a sample size of 5,000 and model 8 at the 95% confidence level. The results showed an indirect effect of competence perception in the self-enhancement situation of 1.28 with a 95% confidence interval (LLCI = 0.615, ULCI = 1.952), which did not contain 0. However, the indirect effect interval for warmth perception was not significant at this point as it contained 0 (LLCI = −0.526, ULCI = 0.744). In the self-transcendence situation, the indirect effect of warmth perception was 0.99 with a 95% confidence interval (LLCI = 0.430, ULCI = 1.569), which did not contain 0; however, the indirect effect interval of competence perception at this point contained 0 (LLCI = −1.055, ULCI = 0.143), which was not significant.

In the same way, we used purchase intention as the dependent variable and showed that in the self-enhancement condition, the indirect effect of competence perception was 0.12 with a 95% confidence interval (LLCI = 0.144, ULCI = 0.212), which did not contain 0; however, the indirect effect interval of warmth perception then contained 0 (LLCI = −0.005, ULCI = 0.356) and was not significant. In the self-transcendence condition, the indirect effect of warmth perception was 0.15 with a 95% confidence interval (LLCI = 0.095, ULCI = 1.410), which did not contain 0. However, the indirect effect interval of competence perception contained 0 (LLCI = −0.089, ULCI = 0.060), which was not significant.

The results of Experiment 2 replicate those of Experiment 1 with a different product category and extend the context to the evaluation of advertising slogans. More importantly, the mediating role of warmth perception and competence perception was verified. For self-transcendence brands, warmth appeal (vs. absence of warmth appeal) increases people’s purchase intention by increasing their perception of warmth; for self-enhancement brands, warmth appeal (vs. absence of warmth appeal) decreases people’s perception of competence, which in turn decreases purchase intention.

## Discussion

Warmth appeals are frequently used in advertising to enhance brand evaluations and brand relationships [e.g., Aaker et al. (2012)]. Previous research has typically supported the positive outcomes of ad warmth (Kervyn et al., 2012). Based on social perception theory, this paper explores boundary conditions and underlying mechanisms of the effectiveness of warmth appeal in advertisements through two experiments. It shows that warmth appeal in advertisements is not always effective. Specifically, for self-enhancement brands, warmth appeal decreases people’s perception of product competence, which in turn decreases positive brand attitude and purchase intention; for self-transcendence brands, warmth appeal increases people’s perception of product warmth, which in turn increases positive brand attitude and purchase intention.

### Theoretical Contribution

This research makes important theoretical contributions. First, it provides a comprehensive understanding of warmth appeal in advertising. Warmth appeal in advertising can stimulate people’s warmth perception and in turn affect such factors as product evaluation and purchase intention. Previous studies have showed that warmth appeal enhances consumers’ evaluations because it brings positive emotional experiences (Pieters et al., 2002; Ang et al., 2007), Our research focusing on boundary conditions for the positive effects of warmth appeal finds that for self-transcendence brands, warmth appeal in advertisements aligns with consumers’ expectations and brings positive effects. This finding extends the view that warmth appeal improves consumer evaluations (Kim and Ball, 2021).

Second, this paper explores underlying mechanisms of the effect of warmth appeal. Previous research has not gone beyond the emotional pathway framework (Aaker and Bruzzone, 1981) to examine how warmth appeal affects consumers’ emotions and hence purchase intention. We investigated how warmth appeal affects warmth perception and competence perception from the perspective of consumers’ reasoning process. More importantly, we focus on how warmth perception and competence perception ultimately influence consumers’ judgments and decisions. Warmth appeal is most effective when it matches the consumer’s perception of the related brand concept. For self-transcendence brands, warmth appeal is consistent with consumers’ expectations, which can bring about a halo effect; for self-enhancement brands, warmth appeal (vs. absence of warmth appeal) is inconsistent with consumers’ expectations, which makes consumers prone to incongruence and thus to deep cognitive processing (Lynch and Schuler, 1994). With such processing, the message of warmth has a stronger reminding effect, and consumers are more likely to associate it with the lack of warmth in the self-enhancement brand, which reduces people’s competence perception. We extend the context of warmth appeal and explore the role of consumers’ assessment of the effectiveness of warmth appeal and test the mediating role of two different perceptions.

Third, this research expands the application of social perception theory. Aaker et al. (2010) included warmth and efficacy to study consumers’ image perceptions of for-profit and non-profit organizations, followed by more studies focusing on their influence on country perception and brand perception (Lynch and Schuler, 1994; Aggarwal, 2004; Torelli et al., 2012a). Our study focuses on how warmth appeal in advertising affects people’s perceptions of warmth and competence. It not only enriches its application to this commercial domain, but also enriches the exploration of antecedents of social perception theory. More importantly, we find that warmth perception and competence perception are determined not by a single factor but by a two-path mechanism. On the one hand, for self-transcendence brands, warmth appeal brings about a halo effect and produces positive effects. On the other hand, for self-enhancement brands, warmth appeal enables consumers to make a compensatory inference between warmth perception and competence perception, which has certain negative effects.

### Managerial Implications

The findings of this paper provide some guidance for companies to improve their communication methods and enhance the effectiveness of marketing communication. Advertising is an important link between brands and consumers, and how to communicate effectively through advertising is an important issue for companies. The influence of warmth appeal in advertising on consumer judgment and decision making is influenced by certain boundary conditions, and in practice, it is important to pay attention to these boundary factors to develop an effective warmth advertising strategy. According to our findings, if a company’s brand concept is entrenched and holds a strong image in consumers’ minds, warmth appeal should be avoided as much as possible to avoid negative inference of competence. If a company has a self-transcendence brand concept, it can use warmth appeal to enhance people’s perceptions.

Second, we explore the relative power of warmth and competence perceptions as underlying mechanisms. We do not absolutely oppose the use of warmth appeal in advertising by companies with a self-enhancement brand concept. To enhance people’s warmth perceptions effectively, they should do so while also minimizing negative inferences about competence.

### Limitations and Future Research

Our research also has some limitations. As the practice of interspersing warmth appeal in advertisements is increasing, whether warmth appeal is integral to a brand is a direction that can be considered in the future. For example, Pepsi’s warmth ads revolve around the Pepsi brand itself, but Budweiser’s warmth ads barely appear with the brand’s own message. Both strategies have brought good results. Thus, it is worth exploring other ways in which warmth appeal influences brand-related outcomes.

Second, a product’s properties are also an important factor influencing the effectiveness of an ad (Dens and De Pelsmacker, 2010). The products used in this study, cars and cameras, both have a high perception of competence. Would similar results be obtained if products with a high perception of warmth (e.g., Soothing Soap) were used? We believe that similar effects would appear because brands have a differentiated positioning and brand image, which determine consumer expectations of the product. The category attributes of the product are also a key factor affecting expectations. However, the influence of brand image on expectations is comprehensive in covering the properties of the product. Thus, as long as warmth appeal in an advertisement is consistent with prior expectations resulting from the brand image, it is likely to produce the results shown in this paper, even with a product with high warmth perception. Of course, a situation in which consumers’ perceived expectations about the product category are too different from the expectations generated by the company’s dominant brand concept cannot be ruled out. Which factor would prevail in the formation of comprehensive consumer expectations needs to be analyzed systematically and in depth in future research.

Third, in this study, we define warmth appeal broadly, but specific emotions could be explored in the future. In practice, the concept of joy is often included, but some highly evocative advertisements that contain negative emotions can also have unexpected effects, such as increasing people’s willingness to share (Akpinar and Berger, 2017). With the boom of social media, future research could consider how different types of advertisements affect people’s sharing behavior.

Finally, in terms of the long-term effects of advertising strategies, we focus on the impact of warmth advertising on consumers’ immediate decision endpoint–judgment and decision making. It is also possible to focus on its long-run effects. Because social perception theory regarding consumers is based on their long-formed expectations from the past, whether matching warmth appeal with expectations can bring long-term effects is an interesting question. In the short term, warmth ads increase the exposure of products and improve brand awareness; for the long term, how to turn warmth ads into high-return winning strategies is worth investigating. Future research can explore the short-term and long-term effects of warmth ads with the help of corporate financial data, which could lead to a more comprehensive understanding of warmth ads.

## Data Availability Statement

The raw data supporting the conclusions of this article will be made available by the authors, without undue reservation.

## Ethics Statement

The studies involving human participants were reviewed and approved by Sichuan University. The patients/participants provided their written informed consent to participate in this study.

## Author Contributions

All authors listed have made a substantial, direct, and intellectual contribution to the work, and approved it for publication.

## Conflict of Interest

The authors declare that the research was conducted in the absence of any commercial or financial relationships that could be construed as a potential conflict of interest.

## Publisher’s Note

All claims expressed in this article are solely those of the authors and do not necessarily represent those of their affiliated organizations, or those of the publisher, the editors and the reviewers. Any product that may be evaluated in this article, or claim that may be made by its manufacturer, is not guaranteed or endorsed by the publisher.
